# Combination of Botulinum Toxin and minocycline Ameliorates Neuropathic Pain Through Antioxidant Stress and Anti-Inflammation via Promoting SIRT1 Pathway

**DOI:** 10.3389/fphar.2020.602417

**Published:** 2021-03-08

**Authors:** Zhi Yu, Jiayu Liu, Le Sun, Yusheng Wang, Hongmei Meng

**Affiliations:** ^1^ Department of Otolaryngology, Bethune First Hospital of Jilin University, Changchun, China; ^2^ Department of Neurology, Bethune First Hospital of Jilin University, Changchun, China

**Keywords:** botulinum toxin, neuropathic pain, SIRT1, anti-inflammation, antioxidant stress

## Abstract

Neuropathic pain (NP) is one of the intractable complications of spinal cord injury (SCI), with poor prognosis and seriously affects the quality of life of patients. This study aims to determine the treatment effect and mechanism of multimodal therapies in a rat model of SCI-induced NP by combining treatment with the anti-inflammatory agent minocycline (MC) and botulinum toxin (BoNT). The combined utilization alleviated SCI-induced NP and reduced apoptosis, inflammation, and oxidative stress of SCI by activating SIRT1 and dampening pAKT, P53, and p-NF-KB. BoNT with a concentration of 0.1 nm and MC with a concentration of 20 uM were selected for the experiment in the primary microglia and astrocytes treated with LPS. It was found that the combination of BoNT and MC obviously inhibits the inflammatory response and oxidative stress of glial cells, and notably activates SIRT1 and restrains pAKT, P53, and p-NF-KB. Therefore, in the treatment of SCI-induced NP, the combination of BoNT and MC markedly improves the therapeutic effect of NP by promoting the SIRT1 expression, thereby inactivating NF-KB, P53, and PI3K/AKT signaling pathway, inhibiting inflammation and oxidative stress as well as relieving SCI-induced NP.

## Introduction

Neuropathic pain (NP) is one of the intractable complications of spinal cord injury (SCI). It is a kind of neuropathic pain with various manifestations that occurs in the area where the skin sensation has disappeared under the injury plane. The incidence is 34%94%, showing an increasing trend in recent years (Zhang et al., 2009; Jonathan et al., 2019). Despite the long-term existence of SCI-induced NP, its pathogenesis is surprisingly limited (David et al., 2018; Palandi et al., 2020). Presently, clinical treatment is limited symptomatically, the effect of which is not ideal, seriously affecting the patients’ quality of life. Therefore, exploring the specific mechanism and possible therapeutic target of SCI-induced NP with effective therapies and alleviating NP pertinently have critical clinical practicability and social benefits.

Botulinum toxin (BoNT) is a kind of bacterial exotoxin produced by *clostridium botulinum*. It combines with cholinergic nerve endings at the neuromuscular junction, inhibits the release of acetylcholine, and relaxes muscles, and is widely used in the treatment of overactive muscle diseases. In recent years, a number of studies have revealed that botulinum toxin can also be used to alleviate pain (Bartley, 2009; Erdocia‐Goni and Hernando de la Bárcena et al., 2019). BoNT has been found to not only to inhibit the release of acetylcholine in synaptic vesicles but also has some influence on the release of other neurotransmitters, including glutamate and substance P. Besides, BoNT injection dramatically reduces or even completely relieves the pain of most patients in the study of dystonia of dorsal neck root. At present, accumulating studies have adopted botulinum toxin to treat various kinds of pain caused by increased muscle tension (Zhang et al., 2019). For example, BTX relieves the pain caused by muscle spasm, and its analgesic effect remains obvious even if the muscle tension returns to normal. Furthermore, BTX is effective for pain caused by both muscle spasm and non muscle spasm, indicating that there are other mechanisms for its analgesic effect. AokiMl et al. found that BTX inhibits inflammatory pain and the release of neurotransmitters such as glutamate and neuropeptides (like substance P) from peripheral sensory nerve endings in formaldehyde-induced inflammatory mice model (Jiangshan et al., 2019). Other studies have also shown that BTX-A acts directly on the nociceptors and makes a great contribution to the pathway of botulinum toxin transferring to the nerve center (Muñoz Lora et al., 2019). Therefore, the function and mechanism of botulinum toxin in SCI-induced NP requires further exploration.

Minocycline (MC) is a semi synthetic tetracycline antibiotic with small molecular weight and strong tissue permeability, which passes through the blood-brain barrier, and has high drug concentration in the brain (Piotrowska et al., 2017; Tang et al., 2019). Previously, minocycline was found to inhibit the activation of microglia, decrease the expression of cyclooxygenase and prostaglandin E2, and reduce the damage to the nerve after ischemia in the transient ischemia model of rats (Zhang et al., 2009). It has been found that minocycline has therapeutic effects on central chronic diseases such as Parkinson's disease, and its effect is related to anti-inflammation. With the development of research, minocycline showed a good analgesic effect in NP (Fatemeh et al., 2019). It is reported that astrocytes are activated in trigeminal neuralgia, while minocycline inhibits the activation of satellite glial cells to attenuate the inflammatory response and alleviate trigeminal neuralgia (Bartley, 2009). Amin et al. reported that tumor necrosis factor-A and IL-1β were highly expressed, while the expression of both was significantly reduced after intraperitoneal injection of minocycline in the model of chronic compression of the sciatic nerve. Zhang et al. found that microglia and its IL-18 signaling pathway are related to mechanical pain, and intrathecal injection of minocycline blocks IL-18 signaling and weakens pain perception. Other researchers also found minocycline reduces the release of glutamate and blocks the sodium channel to relieve pain (Camargos et al., 2020). Therefore, minocycline has a good analgesic effect on NP by attenuating the inflammatory response of glial cells. In addition, botulinum toxin or its degradation products may act on the spinal cord to relieve pain.

There are few studies on the combination of botulinum toxin and microcycline in reducing spinal cord pain. Zychowska Magdalena et al. showed that BoNT/A inhibits the upregulation of IL-18 and IL-1β in CCI-induced spinal cord and/or dorsal root ganglion, but increases the level of IL-10 and IL-1RA, while minocycline enhances the analgesic effect of BoNT/A by inhibiting microglia cells (Magdalena et al., 2016). The combination of botulinum toxin and microcycline greatly improves the efficacy of SCI-induced NP.

Considering that BoNT has effects on inflammatory pain and the release of neurotransmitters and MC has inhibiting effects on microglial activation, this study explored whether combining these two drugs could better relieve the NP. The study aimed to determine the treatment effect and mechanism of multimodal therapies in a rat model of SCI-induced NP by combining treatment with the anti-inflammatory agent minocycline and botulinum toxin.

## Methods

### Establishment of SCI-Induced NP Rats Model

After treatment with isoflurane [2% (v/v); Forane; Abbot, Tokyo, Japan] to induce deep anesthesia, the mice underwent a laminectomy at the T9–10 vertebral level to expose the spinal cord, with a surgical microscope (Vanox; Olympus Optical, Tokyo, Japan). An Infinite Horizon Impactor (Precision Systems and Instrumentation LLC, Fairfax, VA, United States) was adopted to establish a contact SCI model, using an impact force of 60 kdyn. For sham SCI, latency only was performed at T9-T10 without SCI. The round and surrounding skin were cured with 5–0 silk. Forty BALB/c mice (purchased from the Animal Experimental Center of Wuhan University, China) were raised according to standard conditions. All experiments were carried out under the "Guidelines for the Use of Laboratory Animal Care" and supported by the Animal Care Use Committee of the Bethune First Hospital of Jilin University.

### Administration of Minocycline and Botulinum Toxin

Following SCI, mice were assigned randomly to treatment groups. The groups included the sham (without spinal cord injury), SCI (with spinal cord injury and vehicle), SCI + BoNT (with spinal cord injury and BoNT/A treatment), SCI + MC (with spinal cord injury only and MC treatment), and SCI + MC + BoNT (with spinal cord injury only and combining treatment of MC and BoNT/A). To compare the effects of different dosing regimen on antioxidant stress and anti-inflammation, minocycline (25 mg/kg in 0.1 M phosphate buffer; Sigma-Aldrich, St Louis, MO) or vehicle (0.1 M phosphate buffer) was administered via intraperitoneal injection initially at 24 h post-injury, and subsequently every 24 h for 7, 12, and 16 days, at which points mice were sacrificed, respectively. The botulinum toxin received group was injected with botulinum toxin type A (botox A; Allergan, Inc, Irvine, CA) 1 day following injury around the SCI site (5 mm around the injury site with annular injection) and corresponding forelimb muscles (bicep, tricep, brachioradialis, and flexor carpi ulnaris) with two doses of 1.25 U botox A in 0.05 ml saline. Animals that underwent behavioral evaluations received a daily minocycline injection for 2 weeks either by itself or in combination with botox/physical therapy, beginning at 24 h post-SCI. All the injections took place at 10 am.

### Ethology and Pain Assessment in Mice

The Basso Mouse Locomotor Scale (BMS) was used to evaluate locomotor function after thoracic spinal cord contusion or transection injury. It was scored from 0 (hind limb paralysis) to 9 (normal locomotion). BMS scores were recorded at days 0, 3, 7, 14, and 28 post-SCI, and averaged to give one value per mouse.

The Plantar Analgesia Meter for thermal paw (BME-410C, Tianjin, China) was used to measure thermal hyperalgesia, expressed as the thermal withdrawal latency (TWL), namely the time required to notice thermal discomfort and trigger paw withdrawal. Each mouse was measured 3 times, and the average value was taken as the threshold.

Calibrated von Frey fibers (BME-403, Tianjin, China) were used to determine the mechanical withdrawal threshold (MWT) according to the instructions, thereby assessing mechanical hypersensitivity. The measurement was repeated 3 times at an interval of 30 s, and MWT was taken as the mean value. Two independent examiners who were blinded to the experimental results tested the mice at days 0, 3, 7, 14, and 28 post-SCI for TWL and MWT. All the behavioral tests were at 4 pm and with fixed order (BMS, TWL, and MWT).

### Cell Culture and Transfection

Mice astrocytes and microglia were obtained through primary culture using procedures outlined in previous reports (Piotrowska et al., 2017). Briefly, primary cultures of microglia were obtained from mice on postnatal days 1–3. The mouse cerebral cortex was digested with 0.25% trypsin and DNase (Thermo Fisher Scientific, Ma, United States) for 10 min, and cells were passed through a 70 mm nylon mesh. The resultant cell suspension was diluted and seeded into a culture flask. Microglia within the astrocyte monolayer sheet were removed by shaking after 7 days.

Primary astrocytes were obtained from cerebral cortices of 1–3 days old newborn mice. The cortices were mechanically dissociated. The growth medium consisted of DMEM containing 10% heat-inactivated fetal calf serum, and 2 mM glutamine, penicillin (5U/ml, Beyotime Co., Beijing, China), and streptomycin (0.05 mg/ml, Beyotime Co., Beijing, China). Cells were plated in culture flasks and incubated at 37°C in a humidified 5% CO_2_ incubator. After 12–14 days, confluent cultures were shaken for 3 h at 200 rpm, and the medium was discarded. The adherent astrocytes were removed by trypsinization (Beyotime Co., Beijing, China) and re-plated at a density of 10^6^ cells/ml. The secondary cultures consisted of highly purified astrocytes as determined by GFAP staining.

Cells were seeded into the 6-well plate at 5 × 10^6^/well after trypsinization and passage. Cell transfection was conducted after the cell growth was stable. For making the cell injury *in vitro*, LPS (10 ng/ml, Beyotime Co., Beijing, China) was used to treat the microglia or astrocytes for 6 h.

### qRT-PCR

The expression of TNF-a, IL-1 β, IL-6, and IL-8 mRNA in the injured peripheral spinal cord tissues around T9–10 (weight: 1g, volume: 2 ml) was detected by qRT-PCR. Total RNA was extracted with Trizol reagent (invitgen, Carlsbad, CA) according to the manufacturer's guidelines. The mRNA was then transformed into the first strand complementary DNA (cDNA) with the Thermo reverse transcription kit (Thermo Fisher Scientific, Ma, United States). For miRNA analysis, the microRNA first-strand cDNA synthesis package (Sangon Biotech, Shanghai, China) was applied to the reverse transcription process. SYBR Green PCR Master Mix Kit (Applied Biosystems, Foster City, CA) was adopted to amplify TNF-a, IL-1 β, IL-6, and IL-8. All amplifications were carried out in a 7900HT fast real-time system (Applied Biological System). GAPDH served as internal contrast, while the −ΔΔ ^2−ct^ method was used to analyze relative expression. TNF-a: upstream, 5′-AAC​ACG​CGC​TGA​CTC​CTA​GT-3′, downstream, 5′-CAG​TGC​AGG​GTC​CGA​GGT-3′. IL-1β: upstream, 5′-TTC​AAC​CTG​CAT​CCT​ACC​CC-3′, downstream, 5′-GAG​AGA​CAG​ATC​CCG​GAG​AC-3′. IL-6: upstream, 5′-CCC​CGT​AGA​TTG​CAA​ACT​CC-3′, downstream, 5′-TGT​CCT​TGC​CAG​TGT​CTT​CT-3′. IL-8: upstream, 5′-CCC​CGT​AGA​TTG​CAA​ACT​CC-3′, downstream, 5′- TAG​TTC​CTC​TCC​TCT​GGC​CG-3′. GAPDH: upstream, 5′-CGC​TGA​GTA​CGT​CGT​GGA​GTC-3′, downstream, 5′-GCT​GAT​GAT​CTT​GAG​GCT​GTT​GTC-3′.

### 2.6 Western Blot Test

After the animal model was established, the mice were anesthetized according to the experimental schedule. To obtain the injured peripheral tissues in the spinal cord (T9–10) at the dorsal part (weight: 1g, volume: 2 ml), the protein lysate (Thermo Fisher Scientific, Ma, United States) was added to isolate the total protein according to the manufacturer’s instructions. The total protein of 50 μg was separated by 12% polyacrylamide gel electrophoresis at 100 V for 2 h and then electroporated into the PVDF membranes. The membranes were then blocked with 5% skimmed milk and washed with a TBST buffer three times for 10 min each. They were incubated with primary antibodies of SIRT1 (Abcam, ab8805, dilution concentration 1:1000, Ma, United States), Cleaved-caspase-3 (Abcam, ab2302, 1:1000, MA, United States), P53 (Abcam, ab131442, 1:1000, MA, United States), p-P53 (Abcam, ab1431, 1:1000, MA, United States), NF-κB (Abcam, ab32536, 1:1000, MA, United States), p-NF-κB (Abcam, ab86299, 1:1000, MA, United States), AKT (Abcam, ab8805, 1:1000, MA, United States), *p*-AKT (Abcam, ab8933, 1:1000, MA, United States) at 4°C overnight. After being washed with TBST buffer, the membranes were then incubated with horseradish peroxidase (HRP)-labeled secondary antibodies (Abcam, ab6721, diluted concentration 1:1000, Ma, United States) at room temperature for 1 h. The membranes were later washed with TBST 3 times. Finally, a western blot special reagent (Invitrogen Company, United States) was used for color imaging, and ImageJ was used to analyze the gray value of each protein.

### Immunohistochemistry

After paraffin embedding and sectioning (4 μm), the peripheral spinal cord tissues around T9–10 at the dorsal part (weight: 1 g, volume: 2 ml) were dewaxed with xylene, hydrated with gradient alcohol, and inactivated with 3% H_2_O_2_ for 10 min. Microwave repair (pH = 6.0, 15 min) was performed with 0.01 mol/L sodium citrate buffer solution. After being blocked with 5% bovine serum albumin (BSA) for 20 min, the sections were incubated with primary antibodies of SIRT1 (Abcam, ab110304, 1:1000, MA, United States) and Cleaved caspase-3 (Abcam, ab197202, dilution concentration 1:100, Ma, United States) overnight at 4°C. The next day, the secondary antibodies were added respectively and incubated at room temperature for 20 min. DAB color was developed after PBS washing. After counterstaining with hematoxylin, the sections were dehydrated and mount examined under a microscope.

### ELISA (Detection of Oxidative Stress Markers and Inflammatory Response)

The ROS level, SOD activity, and GSH-Px content were determined by ELISA in strict accordance with reagent instructions. Total thiol (TT) was measured with DTNB as a reducing agent. Firstly, 1 ml of tris-EDTA (pH = 8.6) buffer was added to 50 μL of tissue, then the volume was determined at 412 nm by UV spectrophotometry. Subsequently, 20 μL of DTNB reagent (10 mmol/L) was added to the above solution for 15 min, and the concentration was determined according to their ratio. All the above test kits for oxidative stress indicators were purchased from Nanjing Jiancheng Bioengineering Institute.

The peripheral injury spinal cord (T9–10) tissues at the dorsal part were removed and supplemented with 12 times normal saline after being cut up. The tissue and normal saline were then added to the homogenizer, mixed, and crushed fully to obtain tissue homogenate. Finally, the obtained homogenate was centrifuged at 6000r/min at 4°C for 15 min to take the supernatant. The contents of IL-l β, IL-6, and TNF-α were measured according to the manufacturer’s instructions.

### Cell Counting Kit-8 (CCK-8) Assay

The cells of each group were taken, trypsinized, and centrifugated, and then counted before being inoculated into a 96-well plate at 2 × 10^4^/ml with 100 μL per well. After being cultured and treated as previously described in an incubator at 37°C with 5% CO_2_ for 24 h, the medium was discarded. 10 μL of CCK-8 solution was added into each well for incubation for 1 h after treatment according to the experimental groups. The absorbance value of each well was measured at the wavelength of 450 nm by a microplate reader. Four parallel wells were set in each group, and each experiment was repeated 3 times.

### Statistical Treatment

SPSS17.0 (SPSS Inc., Chicago, IL, United States) was used for statistical analysis. The measurement data were expressed by mean ± standard deviation. Pearson correlation test was used for correlation analysis. One-way ANOVA was used for multi-factors comparison. Comparison between the two groups was measured by *t* test. *p* < 0.05 was considered statistically significant.

## Results

### Combination of Botulinum Toxin and Microcycline Alleviated SCI-Induced NP

**FIGURE 1:**
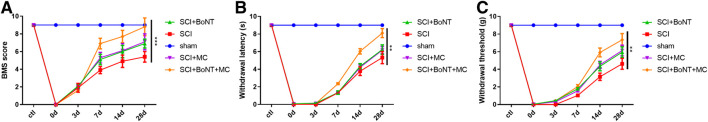
Combination of Botulinum toxin and minocycline alleviated SCI-induced NP. **(A)** BMS score. **(B)** The TWL alteration was detected in the thermal hyperalgesia experiment. **(C)** The MWT alteration was measured in a mechanical hyperalgesia experiment (*p < 0.05, **p < 0.01, ***p < 0.0001, N = 10).

The BMS scores of the mice in the sham group barely changed after SCI and the scores of the SCI group were markedly lower. However, the BMS scores of the mice were significantly increased after the intervention of BoNT and MC, and further enhanced by the combination of BoNT and MC (*p* < 0.05, Figure 1A). The TWL in the sham group didn’t change greatly in the SCI group, which was 3.71 ± 0.49 and 5.17 ± 0.67 s respectively on the 14 and 28th day after SCI. In addition, the TWL was 4.33 ± 0.30 and 6.23 ± 0.54 s after BoNT intervention and was 4.22 ± 0.32 and 6.17 ± 0.40 s after MC intervention. The combination of BoNT and MC further extended the TWL to 6.03 ± 0.30 and 8.10 ± 0.50 s, respectively, (*p* < 0.05, Figure 1B). By contrast, the MWT in the sham group did not change obviously, and it was 3.15 ± 0.41 and 4.62 ± 0.62 g in the SCI group on the 14th and 28th day, respectively. Meanwhile, the MWT was 4.33 ± 0.50 and 6.94 ± 0.60 g after BoNT intervention and was 4.50 ± 0.41 and 6.13 ± 0.57 g, respectively, after MC intervention. The combination of BoNT and MC further extended the MWT to 5.91 ± 0.56g and 7.34 ± 0.72g, respectively, (*p* < 0.05, Figure 1C). These results showed that the combination of BoNT and MC distinctly alleviated SCI-induced NP.

### Combination of BoNT and MC Notably Attenuated Apoptosis, Inflammation, and Oxidative Stress in SCI

**FIGURE 2:**
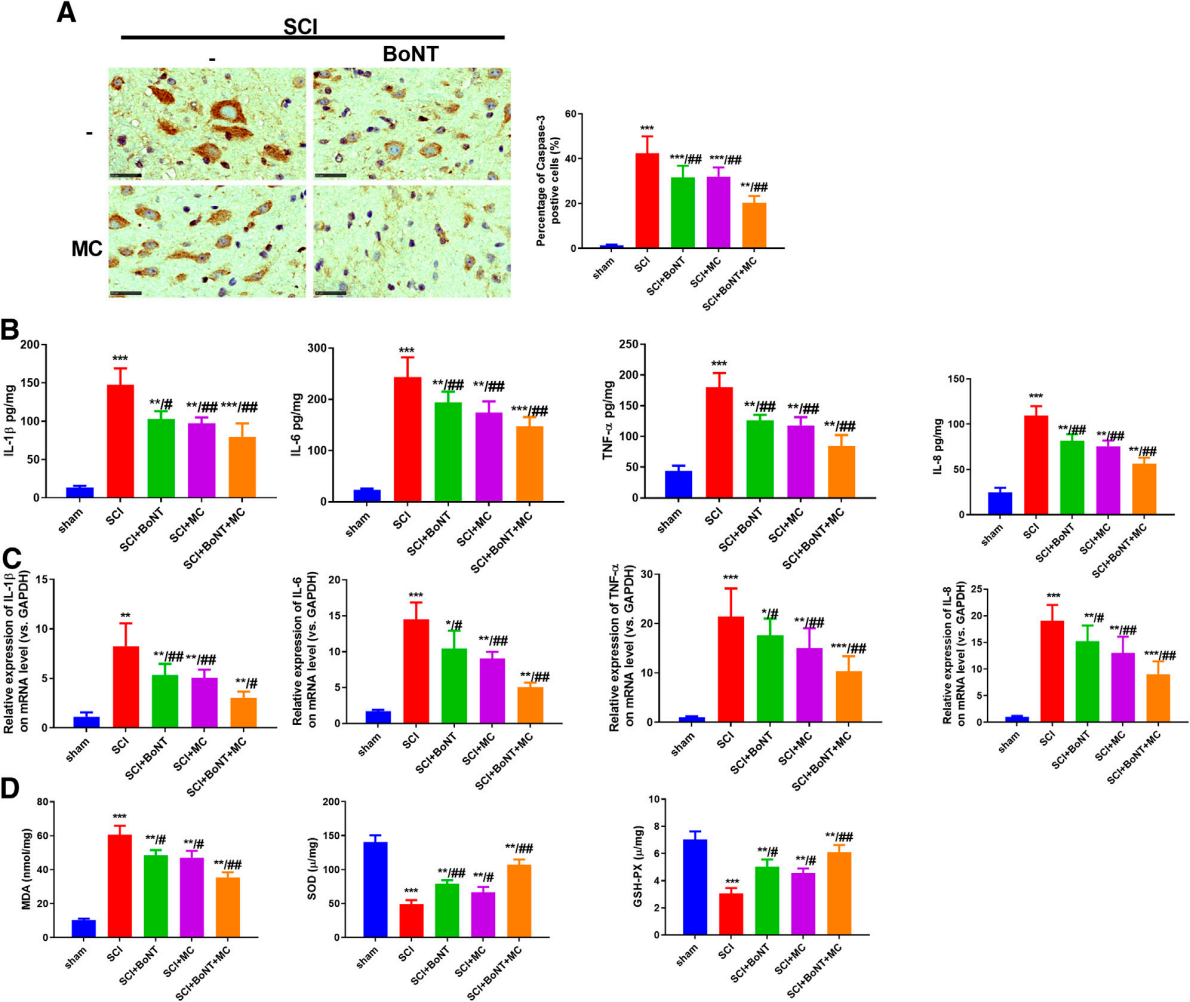
Combination of BoNT and MC greatly reduced apoptosis, inflammation and oxidative stress in SCI. Cappse-3 positive cells in the surrounding tissues of each group were detected by IHC in 14 days after SCI. B-C RT-qPCR and ELISA were adopted to detect alteration of TNF-a, IL-1β, IL-6, and IL-8 at mRNA and protein levels in the peripheral injury spinal cord (T9–10) tissues at the dorsal part in 14 days. D ELISA was used to detect the changes of MDA, SOD, GSH-PX in the injured surrounding tissues in 14 days (*p < 0.05, **p < 0.01, ***P < *p* < 0.0001 V.S sham; ^#^ *p* < 0.05, ^##^ *p* < 0.01, ^###^ *p* < 0.0001 V.S SCI, N = 5).

IHC was used to detect the apoptosis in the injured peripheral tissues 14 days after SCI, while RT-qPCR and ELISA were used to detect the alteration of inflammatory reaction and oxidative stress. The results indicate that Caspase-3 in the injured peripheral tissues was significantly increased 14 days after SCI, and dramatically downregulated after BoNT and MC intervention, and was further weakened by the combination of BoNT and MC (Figure 2A). In contrast, TNF-a, IL-1β, IL-6, and IL-8 were significantly increased in either mRNA or protein levels after SCI, while the level of inflammatory factors was decreased by BoNT and MC intervention, and further reduced by the combination of BoNT and MC (Figure 2B,C). In addition, the results of oxidative stress confirmed that BoNT and MC intervention significantly reduced MDA level, elevated SOD activity, and GSH-PX content, and the combination of BoNT and MC further deepened these effects (Figure 2B). The above results revealed that the combination of BoNT and MC distinctly dampened apoptosis, inflammation, and the oxidative stress damage caused by SCI.

### Combination of BoNT and MC Notably Activated SIRT1 and Inhibited Akt

**FIGURE 3:**
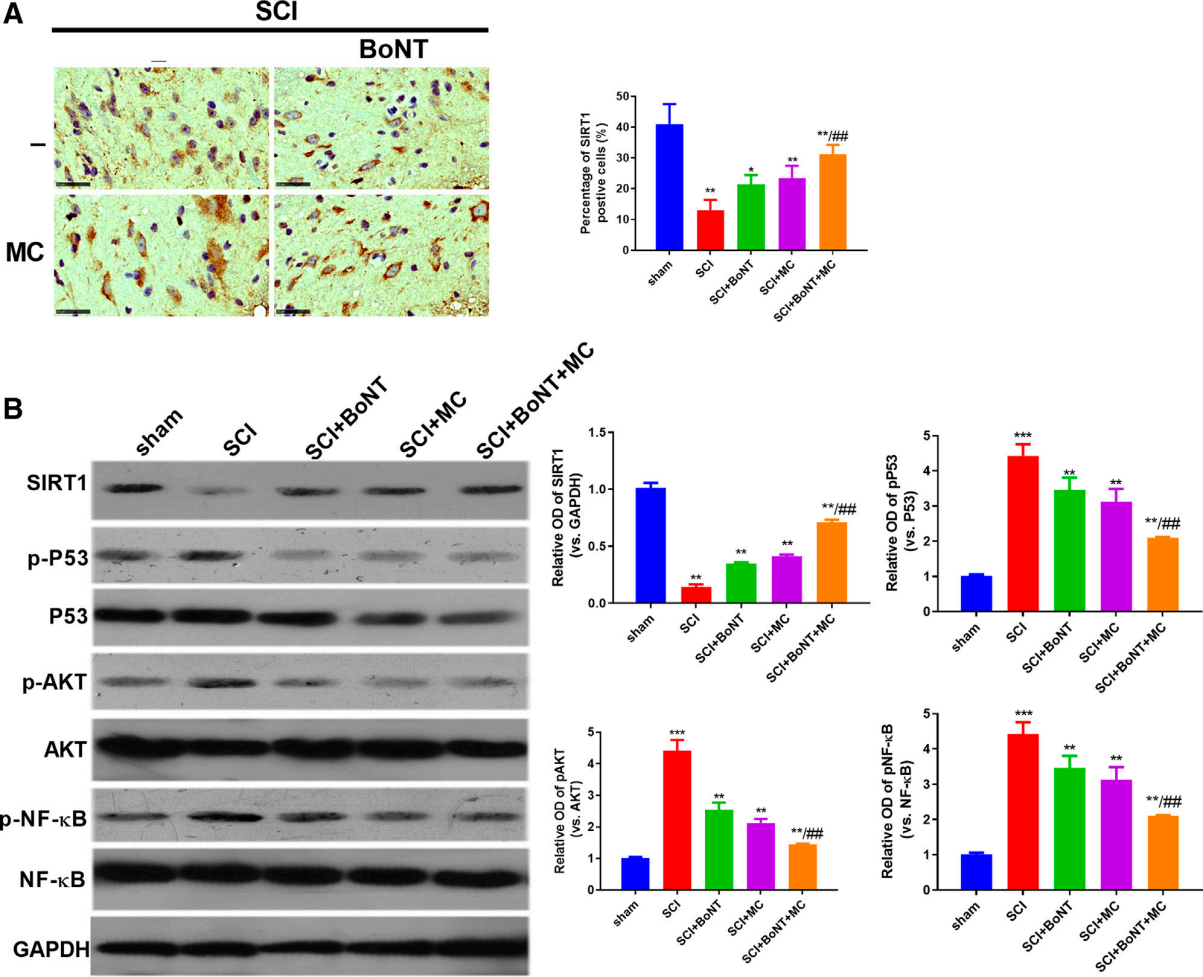
Combination of BoNT and MC dramatically activated SIRT1 and dampened AKT. **(A)** IHC was adopted to detect alteration in SIRT1 expression in injured surrounding tissues after SCI. **(B)** Western blot was used to detect the expression changes of SIRT1, AKT, P53, NF-κB and its phosphorylation after SCI (*p < 0.05, **p < 0.01, ***p < 0.0001 V.S sham; ^#^ *p* < 0.05, ^##^ *p* < 0.01, ^###^ *p* < 0.0001 V.S SCI, N = 5).

To verify the specific mechanism of BoNT and MC combination in alleviating SCI-induced NP, we adopted WB to detect the expressive alteration of SIRT1 and its related signal molecules in peripheral tissues. The results showed that SIRT1 was downregulated, while pAKT, p-P53, p-NF-κB were significantly upregulated after SCI compared with the sham group. In addition, BoNT and MC intervention promoted SIRT1 expression and inactivated AKT, P53, and NF-κB, and BoNT and MC combination further enhanced the above effects (*p* < 0.05, Figure 3A,B). These results suggested that the combination of BoNT and MC notably increased SIRT1 and inhibited AKT, P53, and NF-KB activation.

### Effect of BoNT and MC Combination on the Proliferation of Microglia and Astrocytes

**FIGURE 4:**
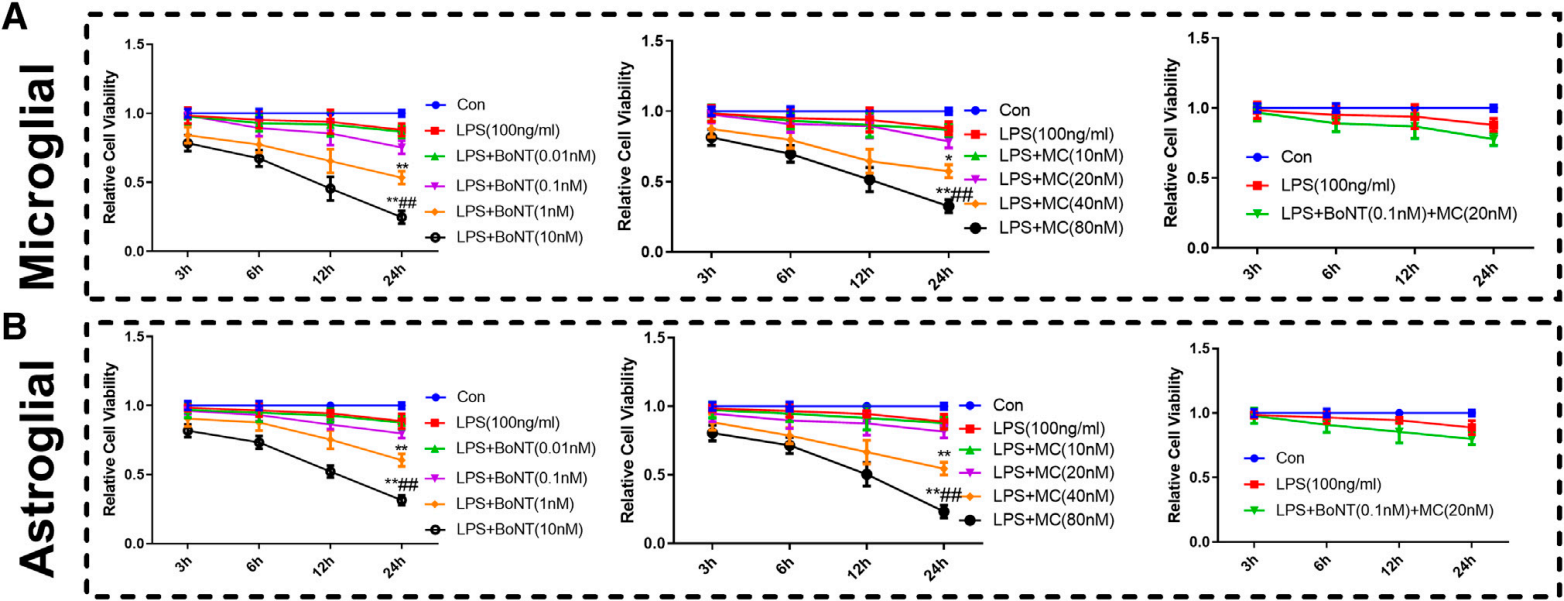
Effect of BoNT and MC combination on the proliferation of microglia and astrocytes. **(A-B)** CCK8 was conducted to detect the effects of BoNT and MC combination on the proliferation of microglia and astrocytes (*p < 0.05, **p < 0.01, ***p < 0.0001 V.S LPS; ^#^ *p* < 0.05, ^##^ *p* < 0.01, ^###^ *p* < 0.0001 V.S LPS + BoNT or and LPS + MC, N = 5).

CCK8 was used to detect the effect of BoNT and MC combination on the proliferation of microglia and astrocytes, and the result revealed that the cell viability of microglia and astrocytes decreased obviously after 24 h of LPS intervention. In addition, different concentrations of BoNT and MC were given, indicating that BoNT with the concentration of 0.01 nm or 0.1 nm had little effect on proliferation, while the BoNT with the concentration of 1 nM or 10 nM distinctly attenuated their proliferation (*p* < 0.05, Figure 4A,B). In contrast, MC with a concentration of 10 um or 20 um had no obvious effect on the proliferation of microglia and astrocytes, while MC with a concentration of 40 um or 80 um markedly weakened proliferation (*p* < 0.05, Figure 4A,B). Furthermore, the effect of BoNT and MC combination on the proliferation of microglia and astrocytes was examined. The combination of BoNT with a concentration of 0.1 nM plus MC with a concentration of 20 um had no remarkable effect on cell proliferation; while that used in other concentrations dramatically dampened cell proliferation (*p* < 0.05, Figure 4A,B). Based on the above results, we selected a combination of BoNT with a concentration of 0.1 nm and MC with a concentration of 20 um as the appropriate concentration in the cell experiment.

### Combination of BoNT and MC Greatly Impeded the Inflammatory Response and Oxidative Stress of Glial Cells

**FIGURE 5:**
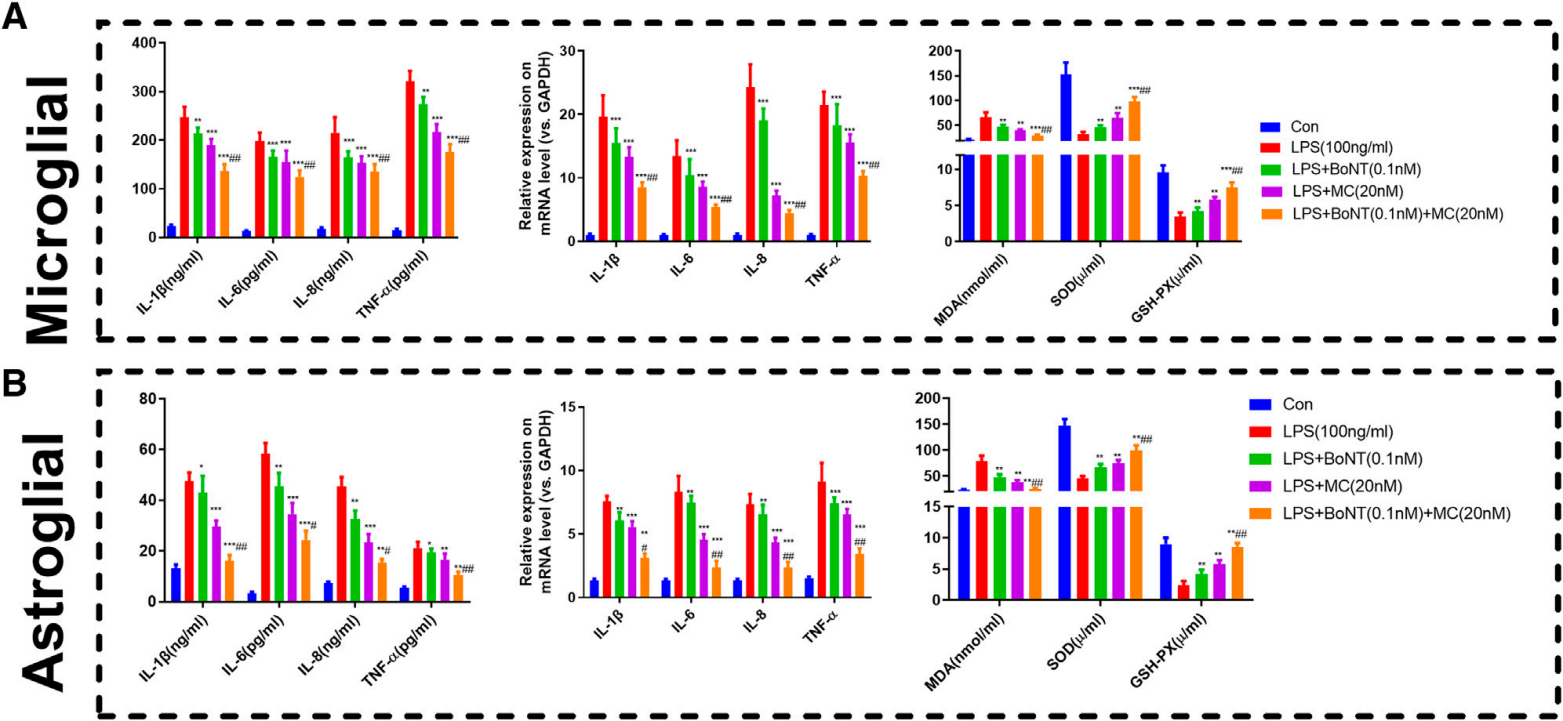
Combination of BoNT and MC distinctly attenuated inflammatory response and oxidative stress in glial cells. **(A-B)** RT-qPCR and ELISA were used to detect alteration in inflammatory response and oxidative stress products (*p < 0.05, **p < 0.01, ***p < 0.0001 V.S LPS; ^#^ *p* < 0.05, ^##^ *p* < 0.01, ^###^ *p* < 0.0001 V.S LPS + BoNT or and LPS + MC, N = 5).

RT-qPCR and ELISA were used to detect alterations of inflammatory response and oxidative stress products. The results suggested that the TNF-a, IL-1 β, IL-6, and IL-8 of microglia and astrocytes in the LPS group were significantly increased in either mRNA or protein level. The level of inflammatory factors was distinctly reduced by BoNT and MC intervention and further impeded by the combination of BoNT and MC (Figure 5A,B). In addition, the results of oxidative stress products showed that BoNT and MC intervention notably reduced MDA level, increased SOD activity, and GSH-Px content, and BoNT and MC combination further strengthened the above effects (Figure 5A,B). The above results verify that a combination of BoNT and MC impedes the inflammatory response of glial cells, while also promoting immune balance and relieving the damage caused by oxidative stress.

### Combination of BoNT and MC Activated SIRT1 and Inhibited Akt of Glial Cells

**FIGURE 6:**
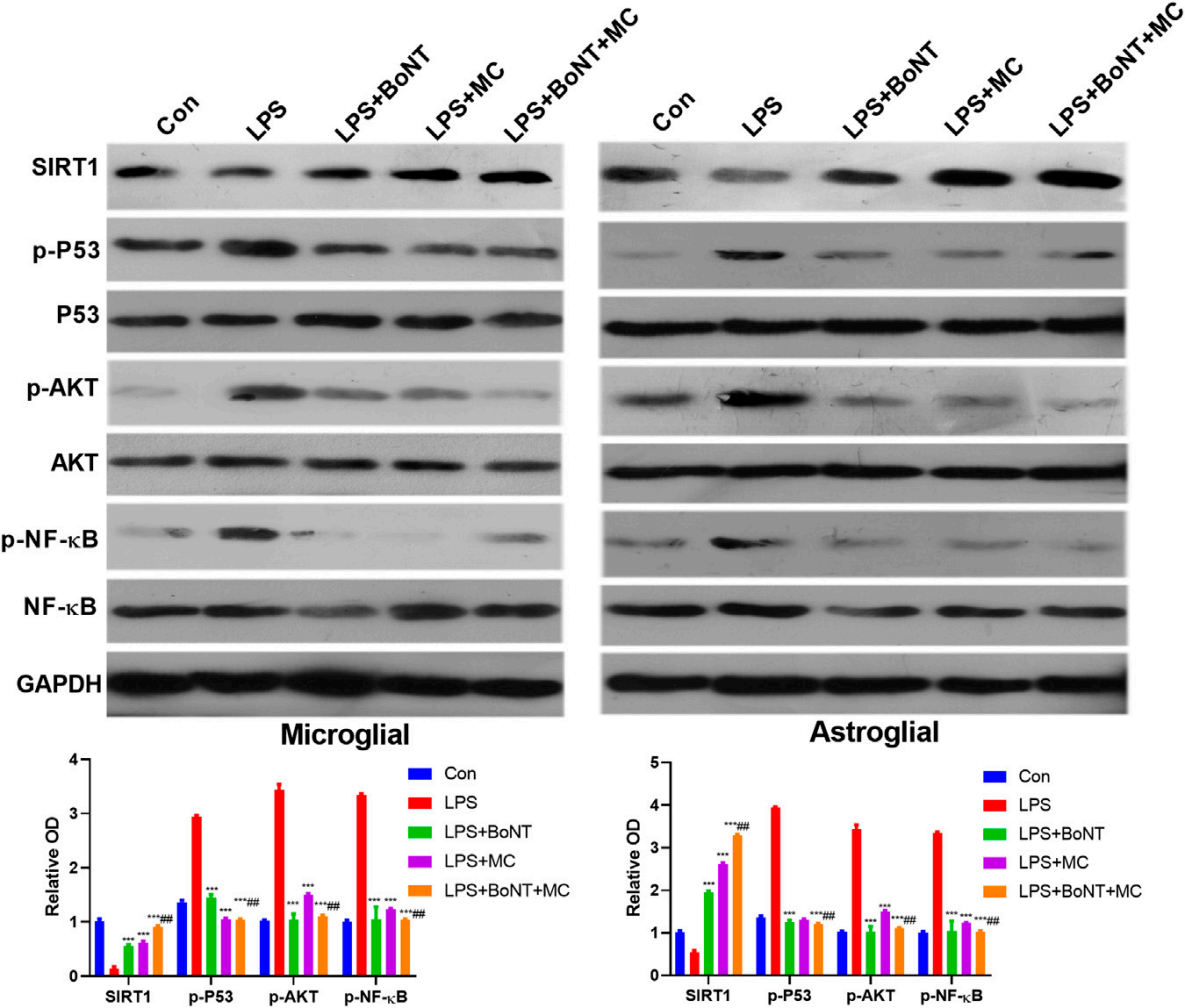
Combination of BoNT and MC dramatically activated SIRT1, inhibited AKT of glial cells. Western blot was used to detect changes in related signaling molecules of SIRT1, AKT, P53, NF-κB and its phosphorylation (*p < 0.05, **p < 0.01, ***p < 0.0001 V.S LPS; ^#^ *p* < 0.05, ^##^ *p* < 0.01, ^###^ *p* < 0.0001 V.S LPS + BoNT or and LPS + MC, N = 5).

To testify the specific mechanism of BoNT and MC in combination, we adopted WB to detect the expressive changes of SIRT1 and its related signal molecules under LPS treatment. The results showed that SIRT1 was significantly downregulated, while pAKT, p-P53, p-NF-κB were significantly upregulated after LPS treatment. Meanwhile, BoNT and MC intervention promoted SIRT1 expression and inactivated AKT, P53, and NF-κB, while BoNT and MC combination further strengthened the above effects (*p* < 0.05, Figure 6A–E). These results suggest that the combination of BoNT and MC dramatically increases SIRT1 and inhibits pAKT, p-P53, and p-NF-KB.

## Discussion

NP is a chronic pain caused by SCI, and a complication of spinal fracture with an incidence of 11%–94% in SCI patients (Zhang et al., 2009; Fatemeh et al., 2019). Cruz Almeida et al. reported that more than 80% of patients with SCI suffered from chronic pain and that 1/3 of them had severe pain. Presently, the treatment of NP is mainly analgesic, which is often ineffective, and the long-term use of painkillers such as opioids causes drug tolerance, dependence, and addiction (Hatch et al., 2018). Exploring new therapies and the mechanisms of NP is an important focus for current pain research. At present, targeted and effective inhibition of cytokines and pain mediators is considered to be a promising way to treat chronic pain (Tang et al., 2019).

A review of the literature by Salat Kinga et al. suggested that botulinum toxin A, minocycline, ambroxol, statins, and PPAR agonists (ATx086001) are expected to be potential novel therapies for NP (Kinga et al., 2018). This study demonstrated that the combined application of BoNT and MC distinctly reduced SCI-induced NP by inactivating glial cells. BoNT was first used in neurogenic detrusor overactivity (NDO) and is a popular drug for treating neuralgia. Dominique Inès et al. reported a total of 107 patients (60.7% with SCI and 36.4% with multiple sclerosis), and BoNT-A seems to be effective and durable for a large number of neurogenic patients after more than 10 years follow-up (Inès et al., 2019). Since most of the causes of NDO are SCI, increasing scholars have reported related research on BoNT in NP, confirming its therapeutic value. Rymkiewicz D et al. injected non-toxic botulinum neurotoxin D (BoNT/D) into mouse primary macrophages. The results showed that BoNT/D is effectively linked to IL-1β ligand (Rymkiewicz et al., 1979). Moon YE et al. reported that BoNT is an effective therapy when conventional therapy cannot alleviate pain (Young Eun et al., 2016). However, in a review of literature, Langevin Pierre et al. found that BoNT-A was not better than normal saline in chronic NP treatment (95% CI-0.50–0.07). BoNT-A combined with rehabilitation exercise and analgesics scarcely reduced chronic NP at 4 weeks. To date, no studies have proved that the use of BoNT-A alone is clinically or statically significant for alleviating chronic NP or subacute/chronic WAD pain (Pierre et al., 2011). On the other hand, Mittal Shivam Om et al. provided effective medical evidence for the efficacy of BoNTs. However, the differences among the most effective injection doses and techniques, optimal dilutions, and BoNT for NP need to be further explored (Mittal Shivam et al., 2016). Thus it can be seen that BoNT has positive therapeutic value in SCI-induced NP, while its effects and methods in clinical application remain elusive. This study suggests that the therapeutic value of BoNT in NP is affirmative, and it may regulate SIRT1 to inactivate glial cells, thereby reducing inflammatory response and oxidative stress, and alleviating NP. This study also suggests that the treatment effect of BoNT alone is average and that increasing the dose may increase the risk of complications. Therefore, we jointly used BoNT and MC to treat SCI-induced NP and evaluate the therapeutic value and mechanism in combination.

Minocycline is a second-generation semi-synthetic tetracycline broad-spectrum antibiotic. It has high lipophilicity, strong tissue penetration, and a good anti-interference effect. It is widely used in the treatment of non-gonococcal urethritis and other infectious diseases. In recent years, with the in-depth study of minocycline, it has been discovered not only to be antimicrobial, but also anti-inflammatory, anti-enzyme, and neuroprotective. A previous report revealed that MC inactivates spinal microglia, but has little effect on astrocytes and neurons. Zhang Qiang, et al. hypothesized in herpes zoster neuralgia (PHN) that minocycline notably inhibits microglial and astrocyte activation and metabolism (Zhang et al., 2009), which has been confirmed in many subsequent studies. Yang Meirong et al. observed that minocycline increases the mechanical pain threshold of neuralgia rats in a dose-dependent manner, and 70 ug of minocycline injection shows a neurotoxic effect. Other studies have found that the injection of 50 ug minocycline increases the mechanical threshold of rats and improves hyperalgesia, with P2X4 receptor downregulation. Nagpal Kalpana et al. used nanoparticle (NP)-coated minocycline hydrochloride (MH) to explore the central anti-nociceptive effect, and found that cMHNP is distinctly central analgesic, demonstrating that the targeted nanoparticles may be used to effectively deliver the central antinociceptive effect of MH (Nagpal et al., 2015). Similarly, minocycline is a semi-synthetic second-generation tetracycline with neuroprotective activity, which effectively passes through the blood-brain barrier. Ahuja Manuj et al. reported that pre-administration of minocycline (50 and 100 mg/kg) prevents 3-NP-induced behavioral dysfunction in a dose-dependent manner. Minocycline also reduces the level of lipid peroxidation and increases the activity of catalase and succinate dehydrogenase. In addition, minocycline downregulates the inflammatory cytokine TNF-α caused by 3-NP and increases the content of catecholamines (dopamine, norepinephrine, and serotonin) in brain homogenates (Ahuja et al., 2008). Shamsi Fatemeh et al. found that minocycline distinctly inhibits the proliferation of neural stem/progenitor cells (NS/PC), but has little effect on cell differentiation (Fatemeh et al., 2019). Minocycline treatment reduces brain damage in the animal cerebral cortex, brain stem, and dampens tumor necrosis factor, interferon-γ, interleukin-6, interleukin-10, and interleukin-12 (Camargos et al., 2020). The above results suggest that MC is an analgesic for NP, and the mechanism may be related to the inhibition of inflammatory factors. This study suggested that MC inhibits the activation of glial cells by weakening inflammation-related signaling pathways, thereby alleviating NP. However, its clinical efficacy and safety remain elusive and need to be further explored by clinical studies.

There is hardly any literature report on the combination of BoNT and MC in NP. However, some scholars have explored the synergistic effect of morphine and minocycline in post-herpetic neuralgia (PHN) and found that MC prevented the development of refractory PHN, weakened the anti-injury tolerance of morphine, and further improved its curative effect. This study suggested that minocycline's selective inhibition of microglial activation reduces the side effects of morphine and improves efficacy (Chen et al., 2010). A study of traumatic brain injury (TBI) showed that minocycline with 25 mg/kg was given 1 day after a craniocerebral injury or sham surgery, once a day for 2 weeks. Botox A was injected in the forearm muscle 1 day after injury, and physical therapy was performed 5 days after injury for 2 weeks. The results showed that inflammation in the cortex and hippocampus around the injury was reduced, but there was no significant difference in the degree of the injury between the groups (Lam et al., 2013). Piotrowska et al. found that BoNT/A and minocycline influence primary microglial cells by inhibiting intracellular signaling pathways, such as p38, ERK1/2, and NF-κB, and pro-inflammatory factors including IL-1β, IL-18, IL-6, and NOS2. Compared with minocycline, BoNT/A did not reduce the release of LPS-induced astrocyte proinflammatory factors, on the contrary, it increased the expression of TLR2 and its adaptor protein MyD88. This result suggests that despite the existence of different molecular targets, the combination of minocycline and BoNT/A dampens the release of microglial-derived pro-inflammatory factors (Piotrowska et al., 2017).

Zychowska Magdalena et al. reported the effects of repeated intraperitoneal injections of minocycline combined with a single injection of BoNT/A on glial cell viability with sciatic nerve chronic compression injury (CCI). The result proved that BoNT/A increases the level of nociceptive factors (IL-10 and IL-1RA) by attenuating the level of injury receptor factors ((IL-1β and IL-1) in dorsal root ganglion, thereby distinctly dampening the pain-related behavior and microglial activation (Magdalena et al., 2016). The current results indicate that the mechanism of combining BoNT and MC is inconsistent and needs to be further explored. It was indicated that the combination of BoNT and MC is positive for the treatment of SCI-induced NP. We further explored new mechanisms and found that the combination of BoNT and MC notably promotes the expression of SIRT1, thereby inhibiting AKT, P53, and other inflammation-related signaling pathways.

SIRT1 belongs to the class of histone deacetylases and has attracted much attention in medical research fields such as diabetes, cardiovascular and cerebrovascular diseases, and tumors. In recent years, scholars have found that SIRT1 inhibits the release of inflammatory factors and reduces damage by inhibiting the NF-κB, P53 and PI3K/AKT signaling pathways, resulting in attenuating 1L-1, IL-6, IL-1β, TNFα, and other inflammatory factors (Li et al., 2018). For example, ING4 dampens NF-κB P65 acetylation and nuclear translocation by targeting SIRT1 and significantly alleviates LPS-induced inflammation and organ damage (Yang et al., 2019). In addition, some studies reported that miR-301a targeted SIRT1, thus attenuating PI3K/AKT and NF-κB signaling pathway (Chen et al., 2017). Junsheng et al. reported that SIRT1 inhibits phosphorylation of Akt in the form of negative feedback (He et al., 2019). This confirms the role of SIRT1 in inhibiting inflammation. The results of this study demonstrate that the combination of BoNT and MC distinctly promote the expression of SIRT1 both *in vivo* and *in vitro*, thus inactivating inflammation and injury-related signaling pathways, such as the NF-κB, P53, and PI3K/AKT.

Another interesting finding was that BoNT and MC improved the BMS score. It seemed that there was no significant difference on day 28 of the experiment. This may provide a promising way of treating dyskinesia, but the mechanism and clinical uses require further exploration.

The combination of BoNT and MC is valuable for the treatment of SCI-induced NP and significantly enhances the therapeutic effect by promoting the expression of SIRT1, thereby inactivating NF-κB, P53, and PI3K/AKT, attenuating inflammatory response and oxidative stress. This study brings novel insights into the potential therapeutic value of the combination of BoNT and MC in NP.

## Data Availability

The raw data supporting the conclusions of this article will be made available by the authors, without undue reservation.
